# New data on spiders (Arachnida, Araneae) of Türkiye, with descriptions of four new species

**DOI:** 10.3897/zookeys.1282.192906

**Published:** 2026-06-19

**Authors:** Alireza Zamani, Yuri M. Marusik, Rahşen S. Kaya

**Affiliations:** 1 Zoological Museum, Department of Biodiversity Sciences, University of Turku, 20500 Turku, Finland Department of Zoology & Entomology, University of the Free State Bloemfontein South Africa; 2 Institute for Biological Problems of the North, FEB RAS, Portovaya Str. 18, Magadan 685000, Russia Institute for Biological Problems of the North, FEB RAS Magadan Russia; 3 Department of Zoology & Entomology, University of the Free State, Bloemfontein 9300, South Africa Faculty of Arts and Science, Bursa Uludağ University Bursa Türkiye; 4 Department of Biology, Faculty of Arts and Science, Bursa Uludağ University, TR-16059, Bursa, Türkiye Zoological Museum, Department of Biodiversity Sciences, University of Turku Turku Finland

**Keywords:** Anatolia, caves, faunistic, intraspecific variation, new record, taxonomy, troglomorphism

## Abstract

New taxonomic and faunistic data on Turkish spiders are presented. Four species are described as new to science: *Cybaeus anatolicus***sp. nov**. (♀; Konya) [Cybaeidae], *Dictyna caligaformis***sp. nov**. (♂♀; Bursa) [Dictynidae], *Leptonetela ayvaensis***sp. nov**. (♂♀; Bursa), and *L. oylatensis***sp. nov**. (♂; Bursa) [Leptonetidae]. Except for *Cybaeus anatolicus***sp. nov**., the remaining new species were collected in caves. Additionally, *Drassodes robatus* Roewer, 1961 [Gnaphosidae] is recorded from Türkiye for the first time, and an unusual variation in the shape of the retrolateral tibial apophysis is documented in an unidentified congener. Additional illustrations are provided for *Cataleptoneta aydintopcui* Demircan, 2020 [Leptonetidae] and *Pterotricha kochi* (O. Pickard-Cambridge, 1872) [Gnaphosidae], for which new localities in Türkiye are reported, as well as for *Troglohyphantes karolianus* Topçu, Türkes & Seyyar, 2008 [Linyphiidae], included here based on topotypic material.

## Introduction

The first checklist of Turkish spiders was compiled by Karol (1967), who summarized published records for 302 species, and was later updated by Bayram (2002) and Topçu et al. (2005). Since then, taxonomic and faunistic studies have increased the known diversity to 1322 species in 374 genera and 58 families (Danışman et al. 2025). Nevertheless, the true diversity of this fauna is likely much higher, as arachnological knowledge of the region remains far from complete due to the scarcity of large-scale surveys. Currently, approximately 215 spider species are considered endemic to this country.

In this paper, we contribute to the knowledge of the spider fauna of Türkiye by describing four new species and reporting one species from the country for the first time. We also provide additional illustrations and new locality records for three species previously known from the country, and document an unusual variation in the shape of the retrolateral tibial apophysis in a species of *Drassodes* Westring, 1851 (Gnaphosidae).

## Material and methods

The material examined in this study was collected from various provinces of Türkiye between 2003 and 2014. Photographs of the specimens and their copulatory organs were taken using an Olympus Camedia E-520 camera mounted on an Olympus SZX16 stereomicroscope at the Zoological Museum of the University of Turku. Digital images captured from multiple focal planes were stacked using Helicon Focus™ 8.1.1. Illustrations of the endogynes were prepared after digesting soft tissues in a 10% aqueous KOH solution. Body measurements exclude the chelicerae and spinnerets. Measurements of palps and legs were taken dorsally and are presented as: total (femur, patella, tibia, metatarsus [absent in palps], tarsus). All measurements are given in millimetres. The number of trichobothria is noted as right-left (r-l) counts for each segment. Spination formula follows the order dorsal-prolateral-retrolateral-ventral, with “p” indicating a pair of spines, following Bolzern et al. (2008, 2009).

### Abbreviations

Male palp: **Av**—anteroventral tibial projection; **Pc**—posterior arm of the conductor; **RTA**—retrolateral tibial apophysis.

Epigyne: **Ac**—anterior chamber; **Cc**—copulatory chamber; **Co**—copulatory opening; **Fd**—fertilization duct; **Ir**—inner receptacle; **Or**—outer receptacle; **Rs**—rounded sclerotization.

### Depositories

**ZMUT**—Zoological Museum of the University of Turku, Finland (V. Vahtera); **ZMUU**—Zoological Museum of Bursa Uludağ University, Türkiye (R.S. Kaya).

## Results

### Family Cybaeidae Banks, 1892

#### Cybaeus anatolicus sp. nov.

Taxon classificationAnimaliaAraneaeCybaeidae

FDE2E981-C0EC-5A03-89D0-E9ADF30579D2

https://zoobank.org/EFB40F5C-4620-4E51-9A7A-405F66563FEB

Fig. 1A–G

##### Type material.

***Holotype***: • ♀ (ZMUT), Türkiye: Konya Prov.: Taşkent-Ermenek plains, 36°50'20"N, 32°34'17"E, 1651 m, 16.5.2012 (R. Kaya). ***Paratypes***: • 1♀ (ZMUT), • 1♀ (ZMUU), collected together with the holotype.

**Figure 1.**
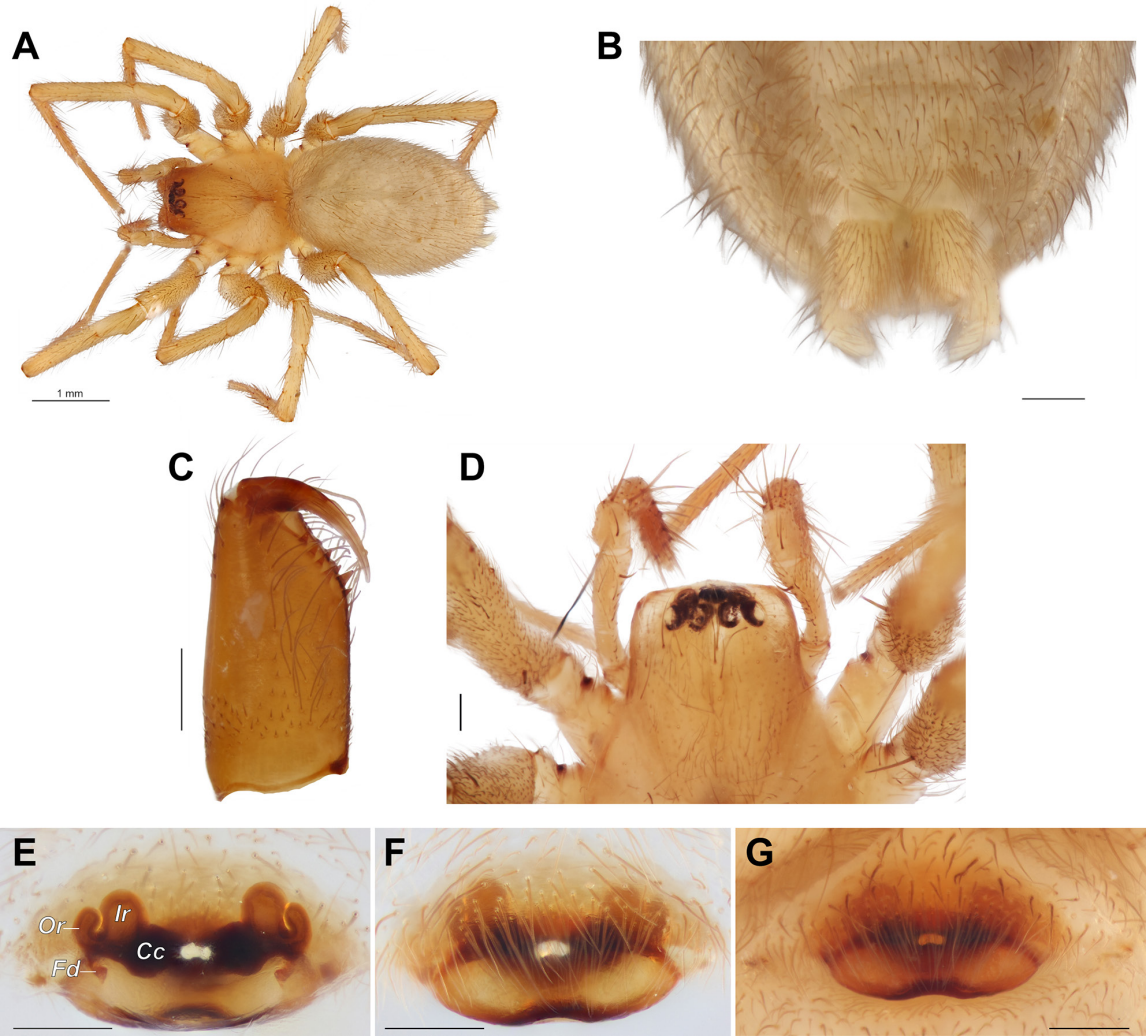
*Cybaeus anatolicus* sp. nov., female. **A**. Habitus, dorsal view; **B**. Spinnerets, ventral view; **C**. Right chelicera; **D**. Anterior part of cephalothorax, dorsal view; **E, F**. Macerated epigyne, dorsal and ventral views; **G**. Intact epigyne, ventral view. Abbreviations: *Cc* = copulatory chamber, *Fd* = fertilization ducts, *Ir* = inner receptacle, *Or* = outer receptacle. Scale bars: 0.2 mm, unless otherwise indicated.

##### Etymology.

The specific epithet refers to the distribution of the species in central Anatolia.

##### Diagnosis.

The new species differs from its congeners by having a transversal copulatory opening (vs. non-transversal), an epigynal plate not divided into two parts (vs. divided), and two pairs of receptacles, each pair located close together and lacking distinct copulatory ducts (vs. two pairs of receptacles connected or separated by copulatory ducts) (cf. Fig. 1E–G vs. Nentwig et al. 2026: figs on the genus page). It also differs in the spination of tibia I, which bears a single pair of ventral spines (vs. at least two pairs of ventral spines; Roth and Brame 1972).

##### Description.

**Female (holotype)**. Habitus as in Fig. 1A. Total length 3.95. Carapace 1.65 long, 1.25 wide. Eyes as in Fig. 1D. Carapace and sternum yellowish brown. Chelicerae, maxillae, and labium pale reddish brown. Chelicerae with 4 pro- and 4 retromarginal teeth (Fig. 1C). Palps and legs yellowish brown, slightly darker distally. Abdomen and spinnerets pale beige (Fig. 1B). Measurements of palp and legs: palp: 2.15 (0.65, 0.35, 0.50, -, 0.65); I: 5.80 (1.65, 0.75, 1.35, 1.30, 0.75); II: 4.90 (1.40, 0.60, 1.05, 1.10, 0.75); III: 4.65 (1.25, 0.60, 0.95, 1.15, 0.70); IV: 6.70 (1.80, 0.70, 1.65, 1.70, 0.85).

Spination: I: Fe 1-2-0-0, Ti 0-0-0-1p, Mt 0-0-0-1p+1p+1p. II: Fe 1-1-0-0, Ti 1-2-0-1+1, Mt 0-2-1-1p+1p+1. III: Fe 1-1-1-0, Ti 1-2-1-1+1p+1p, Mt 1-2-2-1p+1p+1. IV: Fe 1-1-1-0, Ti 1-2-2-1p+1p+1p, Mt 1-3-3-1p+1p+1p. Pa I–IV 2-0-0-0; Ta I–IV 0-0-0-0.

Trichobothria: Mt: I 6-5, II 4-4, III 4-5, IV 2-5; Ta: I 5-5, II 5-4, III 4-3, IV 5-5.

Epigyne as in Fig. 1E–G; epigynal plate ~2× wider than long; fovea small, transversal, posterior part not subdivided; copulatory chambers (*Cc*) heavily sclerotized and wider than inner receptacles; endogyne with 2 pairs of receptacles: relatively large inner receptacles (*Ir*) and smaller, digitiform outer ones (*Or*), almost half as wide as inner receptacles; inner receptacles spaced by ~3× their width; fertilization ducts (*Fd*) originating from outer receptacles.

**Male**. Unknown.

##### Remarks.

The generic placement is tentative and based mainly on similarities in the habitus; the spination and epigyne of the new species clearly differ from those of all other species in Cybaeidae.

##### Distribution.

Known only from the type locality in Konya Province, south-central Türkiye.

### Family Dictynidae O. Pickard-Cambridge, 1871

#### Dictyna caligaformis sp. nov.

Taxon classificationAnimaliaAraneaeDictynidae

D1E0AFE7-1BCE-5F49-8B86-C6E7A31BCDB6

https://zoobank.org/F7ED68CB-9900-4CC9-8734-65BE5F5022F6

**Figure 2.**
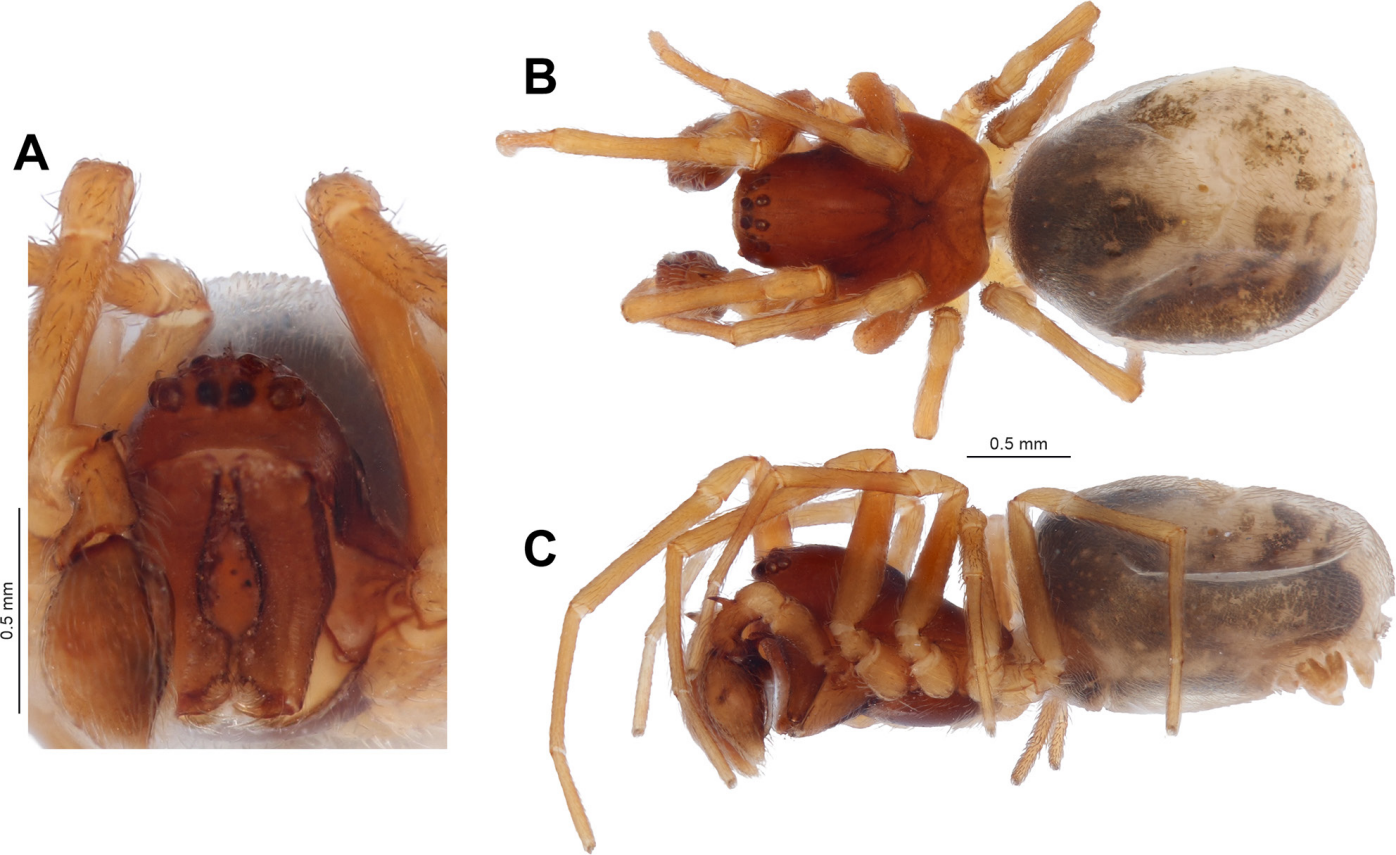
*Dictyna caligaformis* sp. nov., male habitus. **A**. Frontal view; **B**. Dorsal view; **C**. Lateral view.

**Figure 3.**
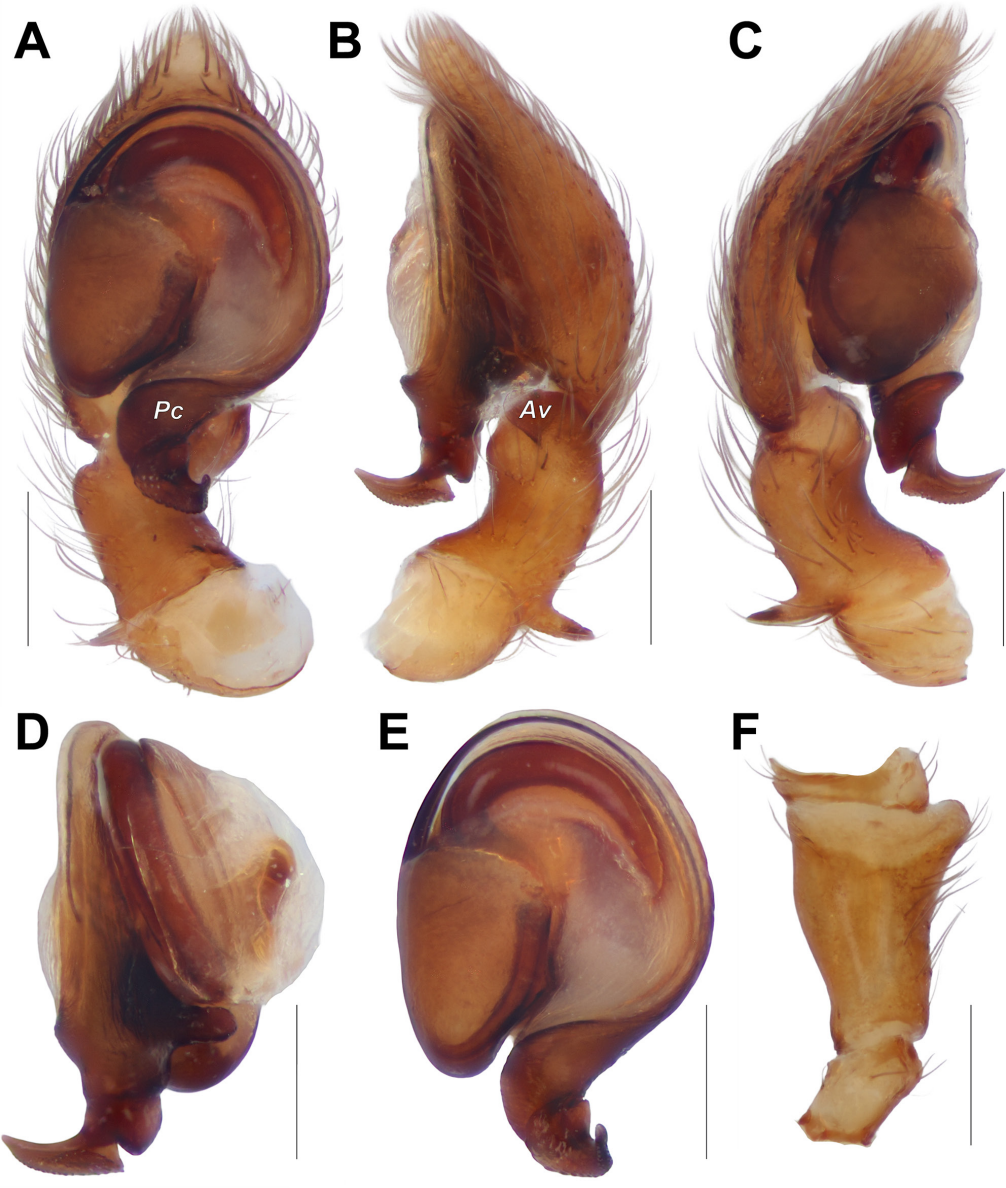
*Dictyna caligaformis* sp. nov., male palp (**A–C, F**) and separated bulb (**D, E**). **A, E**. Ventral view; **B, D**. Retrolateral view; **C**. Prolateral view; **F**. Trochanter, femur, and patella (partial), ventral view. Abbreviations: *Av* = anteroventral tibial projection, *Pc* = posterior arm of the conductor. Scale bars: 0.2 mm.

**Figure 4.**
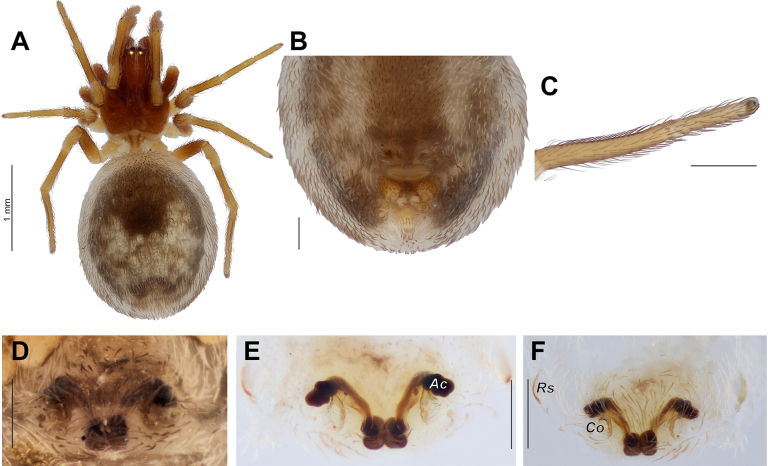
*Dictyna caligaformis* sp. nov., female. **A**. Habitus, dorsal view; **B**. Abdomen, ventral view; **C**. Metatarsus and tarsus IV, lateral view; **D**. Intact epigyne, ventral view; **E, F**. Macerated epigyne, dorsal and ventral views. Abbreviations: *Ac* = anterior chamber; *Co* = copulatory opening; *Rs* = rounded sclerotization. Scale bars: 0.2 mm, unless otherwise indicated.

Figs 2A–C, 3A–F, 4A–F

##### Type material.

***Holotype***: • ♂ (ZMUT), Türkiye: Bursa Prov.: İnegöl Dist., Great Oylat Cave, 39°56'N, 29°35'E, 519 m, 3.6.2009 (Y.M. Marusik). ***Paratypes***: • 2♀ (ZMUT), collected together with the holotype.

##### Etymology.

The specific epithet is derived from *caliga* (Latin for ‘military boot’) and -*formis* (‘shaped like’), referring to the boot-like shape of the posterior part of the conductor in lateral view.

##### Diagnosis.

The new species is similar to *D. uncinata* Thorell, 1856, a species with a trans-Palaearctic distribution, in having a similarly massive posterior arm of the conductor (*Pc*). The male differs from that of *D. uncinata* by the dorsal tibial apophysis shorter than the tibial diameter (vs. as long as the tibial diameter) and by the blunt anteroventral extension of the tibia (vs. conical) (cf. Fig. 3B vs. Roberts 1985: fig. 14d). Females of the two species are almost indistinguishable; however, the new species may be diagnosed by longer anterior chambers (*Ac*) and receptacles, and by the copulatory ducts forming an almost right angle (vs. acute) (cf. Fig. 4E vs. Roberts 1985: fig. 14d).

##### Description.

**Male**. Habitus as in Fig. 2A–C. Total length 3.10. Carapace 1.20 long, 0.95 wide, 0.35 high. Carapace, chelicerae, and labium reddish brown; sternum and maxillae light brown. Palps and legs yellowish brown. Abdomen grey, with indistinct pale beige markings. Spinnerets light brown. Measurements of palp and legs: palp: 1.40 (0.30, 0.25, 0.25, -, 0.60); I: 3.35 (0.95, 0.35, 0.85, 0.70, 0.50); II: 2.90 (0.85, 0.35, 0.65, 0.60, 0.45); III: 1.90 (0.65, 0.30, 0.45, 0.50, 0.30); IV: 2.70 (0.80, 0.30, 0.60, 0.65, 0.35).

Palp as in Fig. 3A–F; tibia ~2× longer than wide, with lobe-shaped anteroventral projection (*Av*); dorsal tibial apophysis shorter than tibial diameter; cymbium ~1.5× longer than wide; anterior arm of conductor terminating at ~10:30 position; posterior arm of conductor (*Pc*) massive, tip bent at right angle; embolus originating at 9 o’clock position.

**Female**. Habitus as in Fig. 4A. Total length 2.95. Carapace 0.95 long, 0.75 wide. Coloration as in male, only slightly paler, and with more distinct abdominal markings. Measurements of palp and legs: palp: 0.95 (0.30, 0.15, 0.20, -, 0.30); I: 2.45 (0.75, 0.30, 0.55, 0.50, 0.35); II: 2.15 (0.70, 0.25, 0.45, 0.45, 0.30); III: 1.90 (0.60, 0.25, 0.40, 0.40, 0.25); IV: 2.40 (0.75, 0.30, 0.50, 0.55, 0.30). Calamistrum uniseriate (Fig. 4C). Cribellum undivided (Fig. 4B).

Epigyne as in Fig. 4D–F; plate without distinct ridges; copulatory openings (*Co*) spaced ~4 diameters apart; copulatory ducts forming almost right angle; anterior chambers (*Ac*) elongated; receptacles contiguous.

##### Remarks.

Rounded sclerotizations (*Rs*) may be homologous to ridges, although they are not connected to the copulatory openings.

##### Distribution.

Known only from the type locality in Bursa Province, northwestern Türkiye.

### Family Gnaphosidae Banks, 1892

#### Drassodes robatus

Taxon classificationAnimaliaAraneaeGnaphosidae

Roewer, 1961

AE0F9CEA-0C4B-5E70-8B38-198D354E765C

Fig. 5A, B

Drassodes robatus Roewer, 1961: 17, figs 28–31, 70 (♂).
Drassodes robatus : Murphy 2007: 55, fig. 446 (♂); Esyunin and Zamani 2019: 69, figs 3A–F, 4A–F (♂♀).

##### Material.

Türkiye: Gaziantep Prov.: • 1♂1♀ (ZMUT), Nizip Dist., 37°00'39"N, 37°37'47"E, 773 m, 11.5.2004 (R. Kaya).

**Figure 5.**
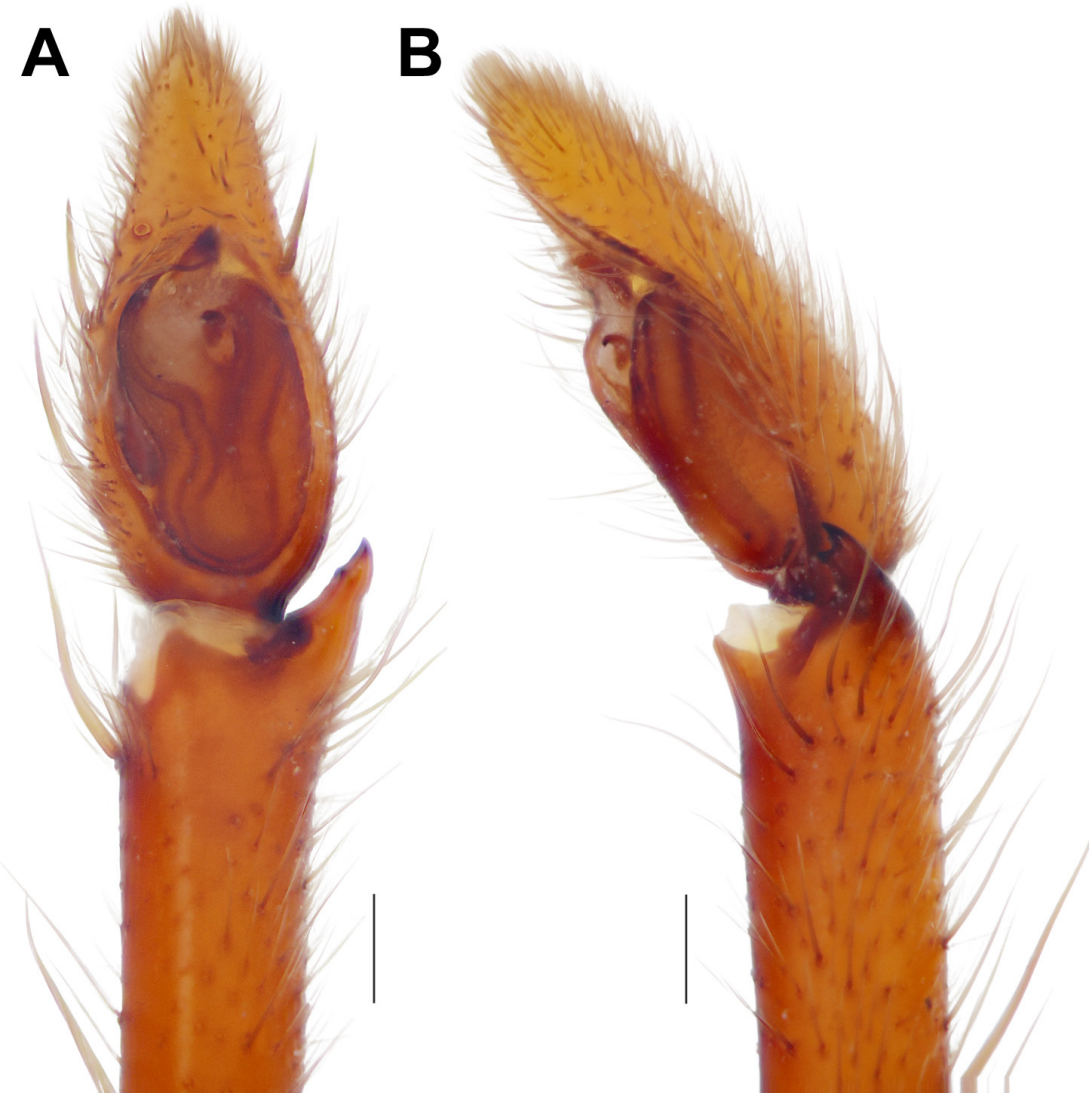
*Drassodes robatus*, male palp. **A**. Ventral view; **B**. Retrolateral view. Scale bars: 0.2 mm.

##### Distribution.

Previously known from Iran and Afghanistan (WSC 2026). New record for Türkiye, with the current material representing the easternmost record of the species in its known range.

#### Drassodes

Taxon classificationAnimaliaAraneaeGnaphosidae

sp.

A36ADF60-6BE6-5BEA-AA46-B56343E43CD1

Fig. 6A–G

##### Material.

Türkiye: Konya Prov.: • 2♂ (ZMUT), Karapınar Dist., Meke Salt Lake, 37°41'37"N, 33°38'20"E, 1030 m, 18.5.2015 (R. Kaya); Isparta Prov.: • 1♂ (ZMUT), Eğirdir Dist., Haymana, 37°34'06.0"N, 30°53'18.0"E, 1010 m, 10.5.2007 (R. Kaya).

**Figure 6.**
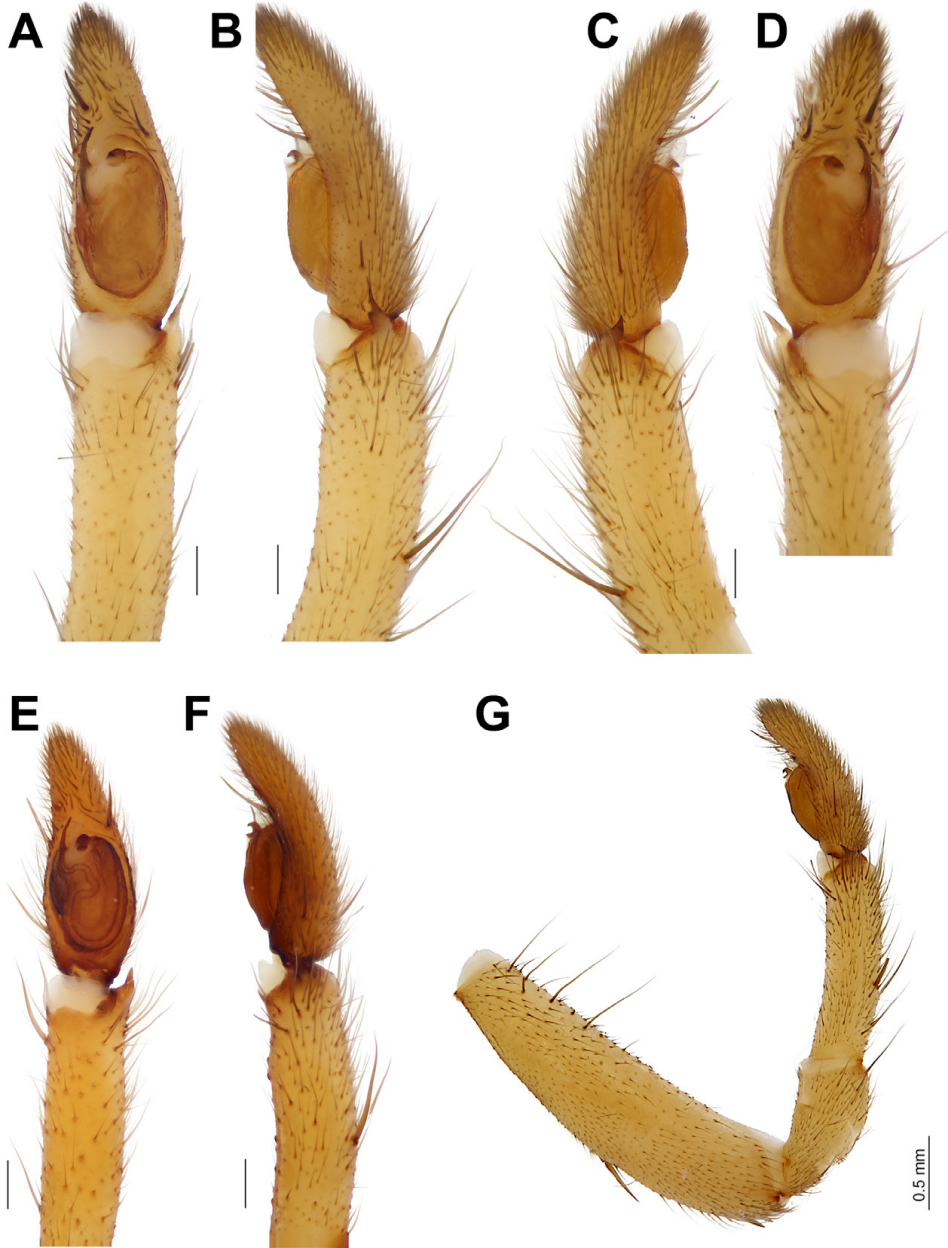
*Drassodes* sp., males from Konya (**A–D, G**) and Isparta (**E, F**), palps. **A, D**. Left and right palp of the same individual, ventral view; **B, C**. Same, retrolateral view; **E, F**. Left palp, ventral and retrolateral views; **G**. Whole palp of another individual, retrolateral view. Scale bars: 0.2 mm, unless otherwise indicated.

##### Remarks.

Considerable variation in the shape of the RTA was observed among the examined specimens, despite their otherwise similar male palp conformation. Variation was even noted between the left and right palps of a single individual from Konya (cf. Fig. 6A, B vs. Fig. 6C, D). The other male collected at the same site in Konya, however, has an RTA shape similar to that of the male from Isparta (cf. Fig. 6G vs. Fig. 6F). Although intraspecific variation in RTA shape has been documented in spiders (e.g., Connolly et al. 2025), differences between the left and right palps of the same individual appear to be rare. Considering these variations, we were unable to identify this species, although it shows similarities to *D. lacertosus* (O. Pickard-Cambridge, 1872) (cf. Fig. 6A–G and Chatzaki and Van Keer 2019: fig. 2).

#### Pterotricha kochi

Taxon classificationAnimaliaAraneaeGnaphosidae

(O. Pickard-Cambridge, 1872)

B9357846-C94C-52DF-803B-F245D0E4EAF2

Fig. 7A–F

Pterotricha kochii: Levy 1995: 967, figs 121–125 (♂♀).^1^

##### Material.

Türkiye: Konya Prov.: • 1♂1♀ (ZMUT), Karapınar Dist., Meke Salt Lake, 37°41'37"N, 33°38'20"E, 1030 m, 18.5.2015 (R. Kaya).

**Figure 7.**
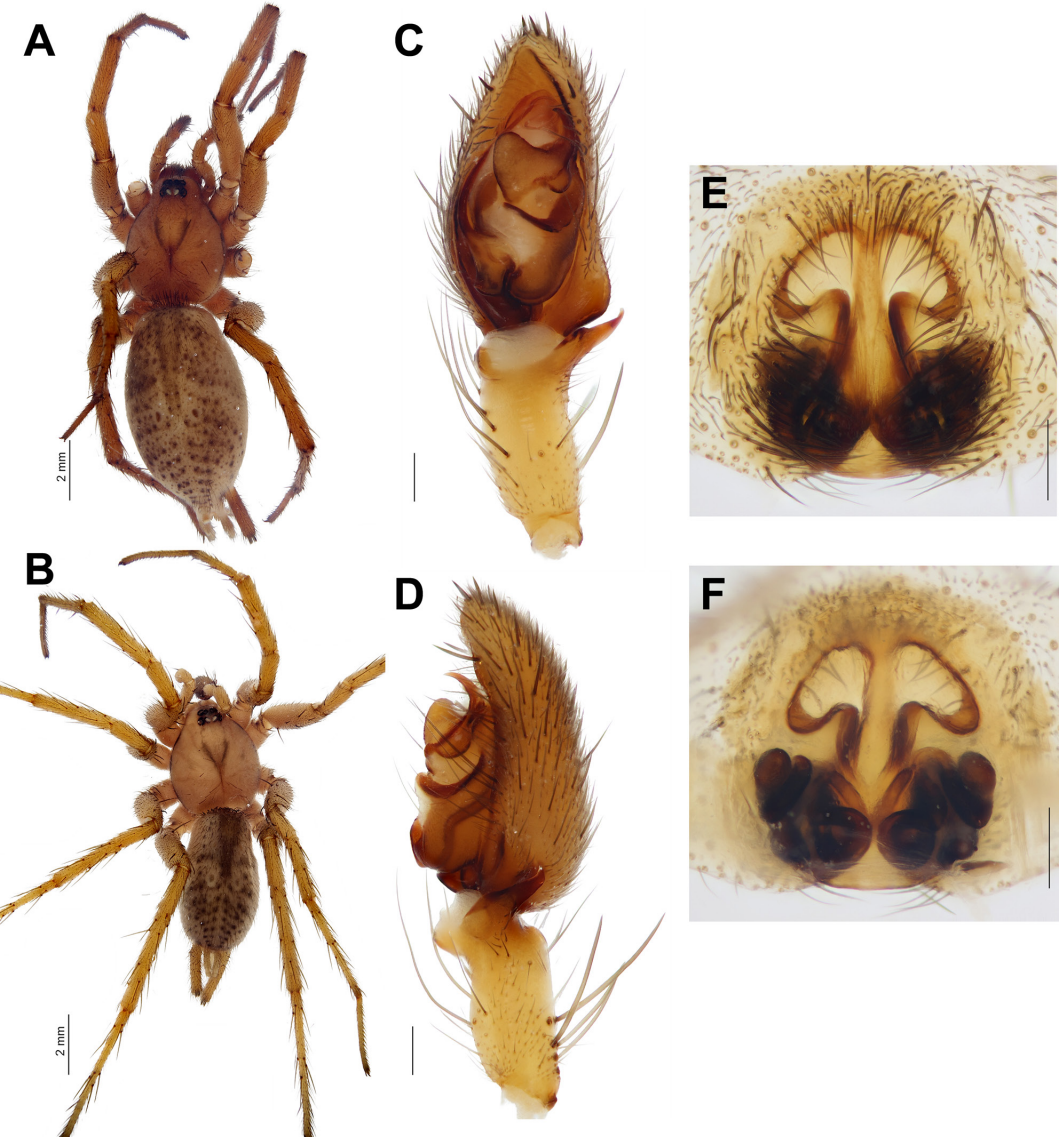
*Pterotricha kochi*, female (**A, E, F**) and male (**B–D**). **A, B**. Habitus, dorsal view; **C, D**. Palp, ventral and retrolateral views; **E, F**. Macerated epigyne, ventral and dorsal views. Scale bars: 0.2 mm, unless otherwise indicated.

##### Remarks.

All illustrations for this species in the literature are drawings; here, we illustrate the habitus for the first time and provide photographs of the copulatory organs.

##### Distribution.

Türkiye to the Levant (WSC 2026). In Türkiye, it was previously known from Konya (Nosek 1905, Dalmas 1921), Gaziantep, Osmaniye, Hatay (Kumbiçak and Kumbiçak 2014), and Kütahya (Seyyar et al. 2019) provinces, southern to central-western Türkiye.

### Family Leptonetidae Simon, 1890

#### Cataleptoneta aydintopcui

Taxon classificationAnimaliaAraneaeLeptonetidae

Demircan, 2020

3F9EDFF0-4288-535A-9041-B32A1D4D82C2

Fig. 8B, E

Cataleptoneta aydintopcui Demircan, 2020: 188, figs 1A, B, 2A–E, 3A–C (♂♀).

##### Material.

Türkiye: Antalya Prov.: • 2♂1♀ (ZMUT), Döşemealtı Dist., Yağca Vill., Karain Cave, 37°04'40.0"N, 30°34'15.0"E, 4.8.2009 (R. Kaya, C. Kaya).

**Figure 8.**
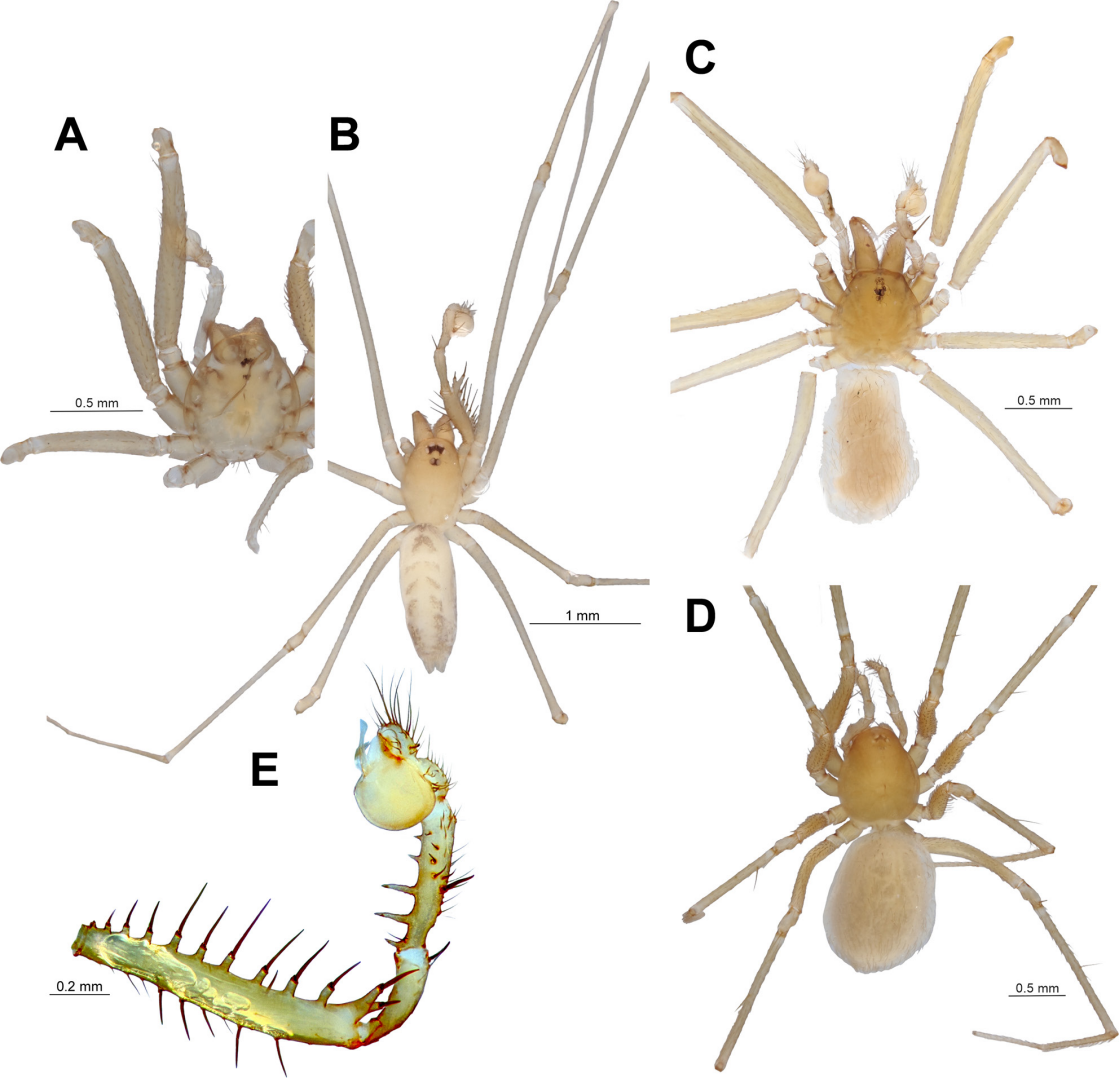
*Leptonetela ayvaensis* sp. nov. (**A, D**), *Cataleptoneta aydintopcui* (**B, E**) and *Leptonetela oylatensis* sp. nov. (**C**), males (**A–C, E**) and female (**D**). **A–D**. Habitus, dorsal view; **E**. Whole palp, retrolateral view.

##### Distribution.

Previously known only from the type locality in Mersin Province, northwestern Türkiye (Demircan 2020). The new locality reported here extends the range of the species approximately 320 km eastwards.

#### Genus Leptonetela

Taxon classificationAnimaliaAraneaeLeptonetidae

Kratochvíl, 1978

4935345D-8E01-55E0-8867-672E4F462103

##### Remarks.

This genus currently comprises 124 described species distributed from Greece to China (WSC 2026), including two from Türkiye: *L. deltshevi* (Brignoli, 1979) (♂; Ordu Province, northern-central Türkiye) and *L. turcica* Danışman & Coşar, 2021 (♂♀; Kahramanmaraş Province, southern-central Türkiye).

#### Leptonetela ayvaensis sp. nov.

Taxon classificationAnimaliaAraneaeLeptonetidae

A7ED9E2C-8D84-56F1-9183-2AB3B1E798D2

https://zoobank.org/9B16248C-FCCE-461A-B39B-F0ED79CF770F

Figs 8A, 8D, 9A–F

##### Type material.

***Holotype***: • ♂ (ZMUT), Türkiye: Bursa Prov.: Nilüfer Dist., Ayvaini Cave, 40°07'27.0"N, 28°42'03.6"E, 12.11.2012 (R. Kaya). ***Paratype***: • 1♀ (ZMUT), collected together with the holotype.

**Figure 9.**
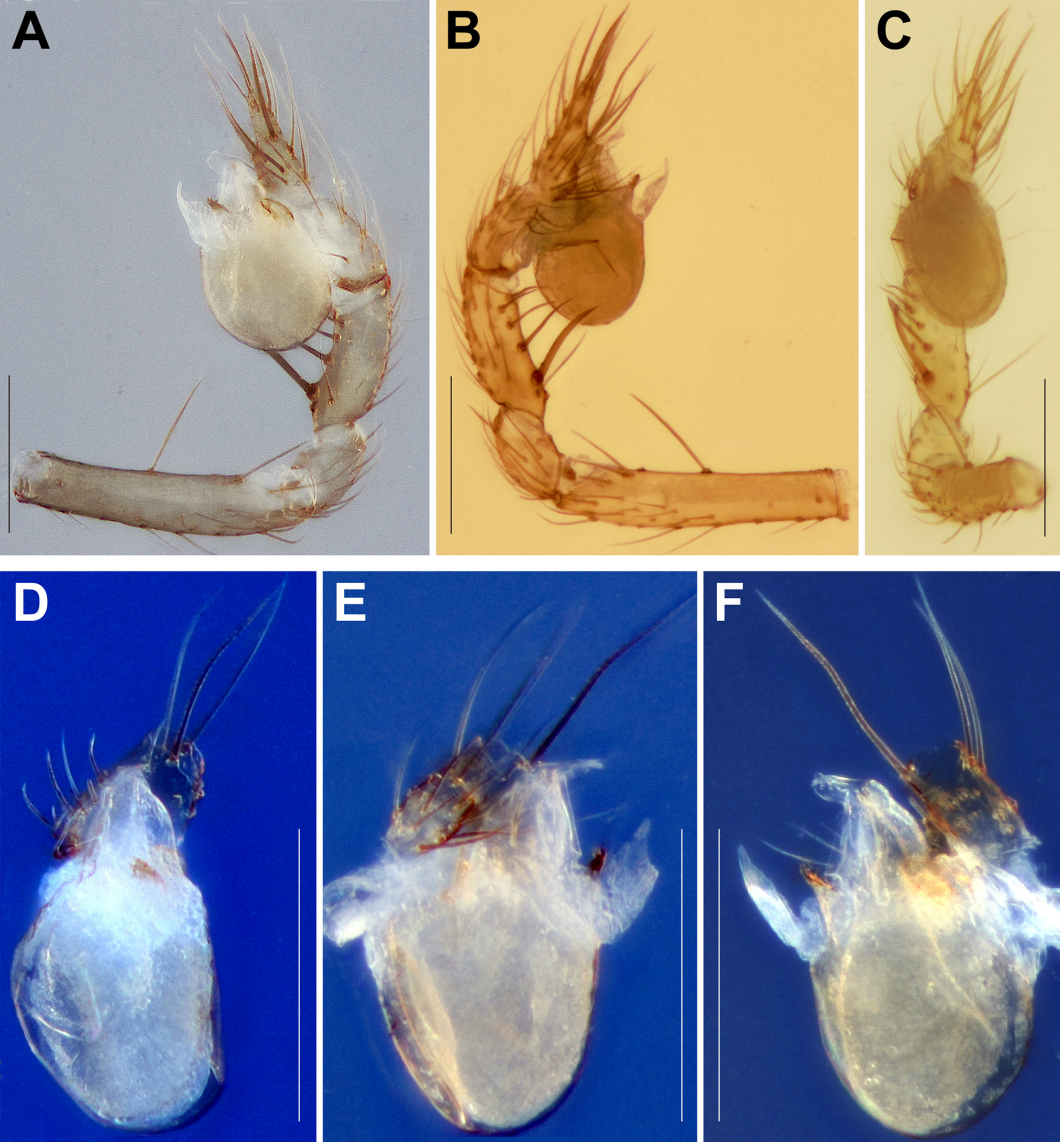
*Leptonetela ayvaensis* sp. nov., male palp (**A–C**) and separated bulb (**D–F**). **A, F**. Retrolateral view; **B, E**. Prolateral view; **C, D**. Ventral view. Scale bars: 0.2 mm.

##### Etymology.

The specific epithet refers to the type locality of the species.

##### Diagnosis.

The new species is similar to *L. deltshevi* in having the same number and shape of prolateral spines on the palpal tibia, but can be distinguished by its smaller size (carapace 0.65 vs. 0.78 mm), a shorter femur (length/width ratio 5.2 vs. 6), and tibia lacking a ventrobasal bulge (cf. Fig. 9A vs. Brignoli 1979: fig. 2).

##### Description.

**Male**. Habitus as in Fig. 8A. Carapace 0.65 long, 0.60 wide. Specimen in poor condition; abdomen and most leg segments missing. Measurements of palp and femora and patellae of legs I–III: palp: 1.05 (0.40, 0.20, 0.15, -, 0.30); I: 1.25, 0.25; II: 1.15, 0.20; III: 0.90, 0.20.

Palp as in Fig. 9A–F; femur 5.2× longer than wide, with strong ventral seta in middle; patella 3× shorter than femur; tibia almost 2× shorter than femur, with 5 strong prolateral spines, proximal spines much stronger than others; anterior part of cymbium with long ventral setae and setae at tip; bulb ~1.6× longer than wide; terminal part with conical sclerite bearing 4 teeth and 2 membranous lamellae.

**Female**. Habitus as in Fig. 8D. Total length 2.10. Carapace 0.80 long, 0.70 wide. Cephalothorax and its appendages pale yellowish brown. Abdomen and spinnerets pale beige. Measurements of palp and legs: palp: 1.35 (0.45, 0.15, 0.30, -, 0.45); I: 4.60 (1.30, 0.30, 1.30, 0.95, 0.75); II: 4.05 (1.15, 0.25, 1.10, 0.90, 0.65); III: 3.40 (0.95, 0.25, 0.85, 0.80, 0.55); IV: 4.55 (1.25, 0.25, 1.30, 1.05, 0.70).

Endogyne. Lost before photography.

##### Distribution.

Known only from the type locality in Bursa Province, northwestern Türkiye.

#### Leptonetela oylatensis sp. nov.

Taxon classificationAnimaliaAraneaeLeptonetidae

88412DFA-CCAB-5D90-882A-747D4C329B77

https://zoobank.org/A08835FB-B8F4-48B4-977E-4C2DAB0DAD6B

Figs 8C, 10A–F

##### Type material.

***Holotype***: • ♂ (ZMUT), Türkiye: Bursa Prov.: İnegöl Dist., Great Oylat Cave, 39°56'62.0"N, 29°35'49.0"E, 22.7.2003 (R. Kaya).

**Figure 10.**
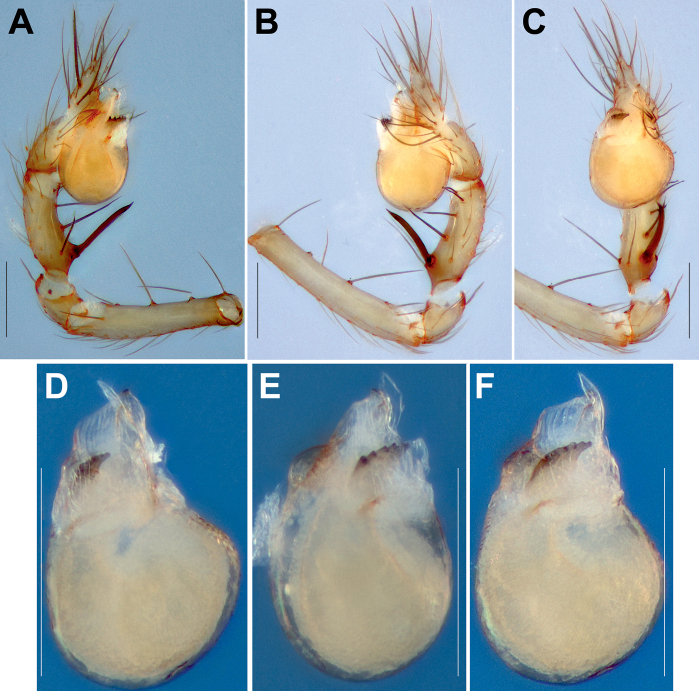
*Leptonetela oylatensis* sp. nov., male palp (**A–C**) and separated bulb (**D–F**). **A, E**. Prolateral view; **B, D**. Retrolateral view; **C, F**. Ventral view. Scale bars: 0.2 mm.

##### Etymology.

The specific epithet refers to the type locality of the species.

##### Diagnosis.

The new species is similar to *L. turcica* Danışman & Coşar, 2021 in having a very strong ventral spine on the male palpal tibia, but can be distinguished by the spine being acute rather than widened at the tip (cf. Fig. 10B vs. Danışman and Coşar 2021: fig. 10B). The new species also differs in having ventral femoral setae, which are absent in the sibling species, and a longer femur I (1.45 vs. 1.0).

##### Description.

**Male**. Habitus as in Fig. 8C. Total length 1.85. Carapace 0.70 long, 0.65 wide. Cephalothorax yellowish brown. Palps and legs pale yellowish brown. Abdomen and spinnerets pale beige. Measurements of palp and legs (I, II and IV only femora and patellae): palp: 1.15 (0.45, 0.15, 0.25, -, 0.30); I: 1.45, 0.30; II: 1.25, 0.25; III: 3.30+missing tarsus (1.15, 0.20, 1.05, 0.90, missing); IV: 1.40, 0.25.

Palp as in Fig. 10A–F; femur long, length/width ratio 6.3, slightly longer than patella + tibia, venter with 4 long setae; tibia ~4× longer than wide, with very strong ventro-basal spine and 4 setae; cymbium slightly longer than tibia, anterior half with long straight setae; bulb ~1.5× longer than wide, terminal part with ridge-like sclerite bearing 6 teeth and 2 membranous lamellae.

**Female**. Unknown.

##### Distribution.

Known only from the type locality in Bursa Province, north-western Türkiye.

### Family Linyphiidae Blackwall, 1859

#### Genus Troglohyphantes

Taxon classificationAnimaliaAraneaeLinyphiidae

Joseph, 1882

286BA56B-E02C-58ED-9FB7-CA803A7858E0

##### Remarks.

*Troglohyphantes* is a large genus of Micronetinae comprising 139 described species. Of these, four species are known from Türkiye: *T. gladius* Wunderlich, 1995 (♂; Ordu Province, northern-central Türkiye); *T. karolianus* Topçu, Türkes & Seyyar, 2008 (♂♀; Konya Province, central Türkiye); *T. pisidicus* Brignoli, 1971 (♀; Isparta Province, southwestern Türkiye); and *T. turcicus* Topçu, Türkeş, Seyyar, Demircan & Karabulut, 2014 (♂♀; Bilecik Province, northwestern Türkiye).

#### Troglohyphantes karolianus

Taxon classificationAnimaliaAraneaeLinyphiidae

Topçu, Türkes & Seyyar, 2008

C510F96F-8FE1-5834-8648-44ED8A3261CA

Fig. 11A–E

Troglohyphantes karolianus Topçu et al., 2008: 91, figs 1, 2, 3ab, 4a–c, 5a–c (♂♀).

##### Material.

Türkiye: Konya Prov.: • 2♀ (ZMUT), Beyşehir Dist., Kurucaova Vill., İnönüini Cave, 37°40'N, 31°21'E, 1200 m, 19.5.2010 (R. Kaya).

**Figure 11.**
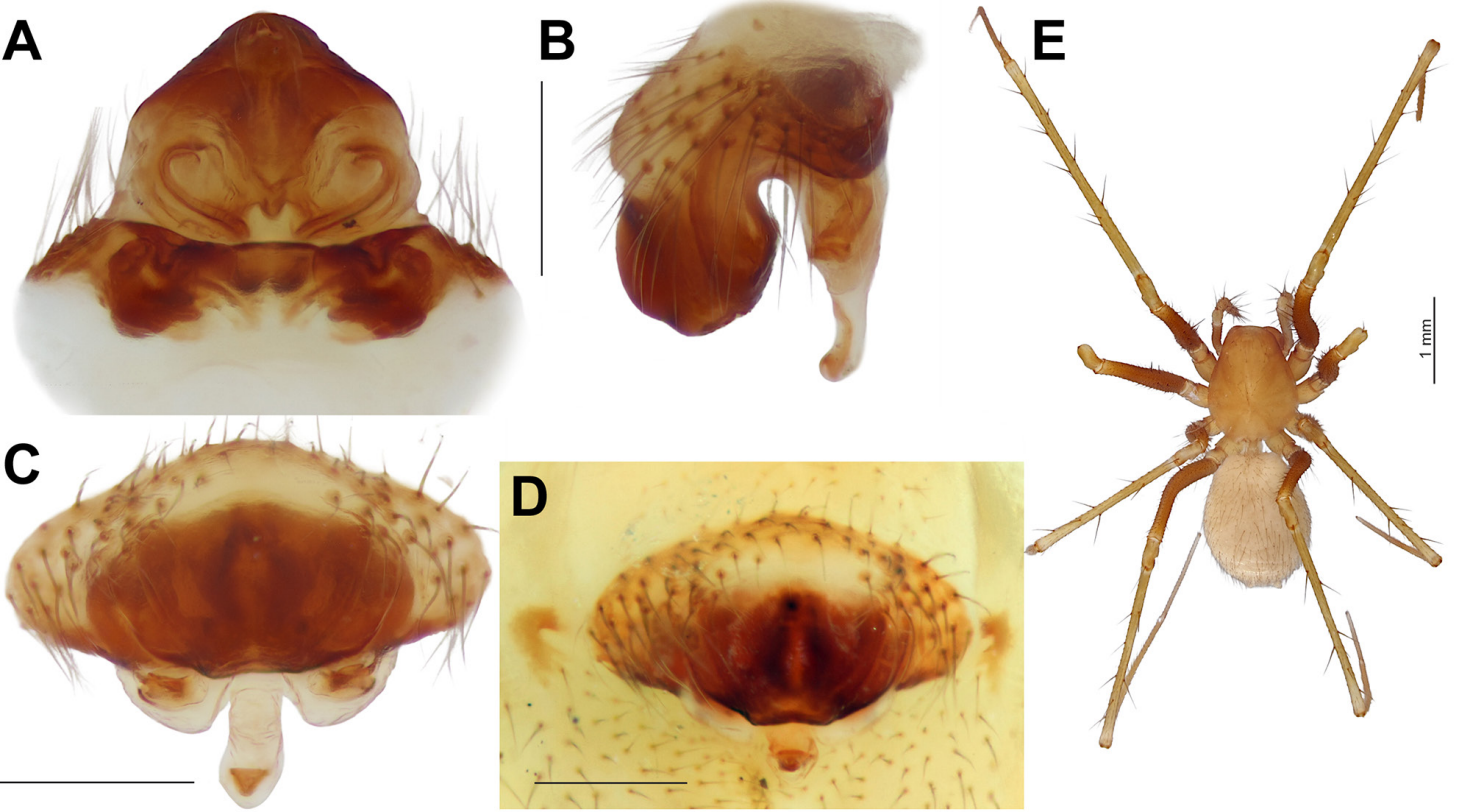
*Troglohyphantes karolianus*, female. **A–C**. Macerated epigyne, posterior, lateral, and ventral views; **D**. Intact epigyne, ventral view; **E**. Habitus, dorsal view. Scale bars: 0.2 mm, unless otherwise indicated.

##### Remarks.

Here, we present, for the first time, details of the epigyne of this species, including posterior and lateral views, as well as an image of its habitus. In their original description, Topçu et al. (2008) considered *T. polyophthalmus* Joseph, 1882, known only from Slovenia, to be the most similar species. However, *T. karolianus* appears to be more closely similar to *T. pisidicus* Brignoli, 1971, known only from two females collected in Hacı Akif Cave, Konya Province (Brignoli 1971), merely 10 km from the type locality of *T. karolianus*. The two nominal species may in fact be synonymous; however, *T. karolianus* can tentatively be distinguished from *T. pisidicus* by a longer ridge on the ventral epigynal plate (cf. Fig. 11C vs. Brignoli 1971: fig. 1) and by its larger overall size (carapace 1.1–1.2 mm vs. 0.85 mm).

##### Distribution.

Known only from the type locality in Konya Province, central Türkiye (Topçu et al. 2008; present data).

## Discussion

Approximately 35,000–40,000 caves occur in Türkiye’s karstic regions (MTA 2024), most of which remain largely unexplored regarding their invertebrate fauna. The earliest studies on Turkish cave spiders were conducted by Roewer (1959), Brignoli (1968, 1971, 1972, 1978a, 1978b), and Gasparo (2007), who collectively described 25 species new to science and recorded an additional 70 species from these caves. Subsequent contributions include Kunt et al. (2008), who studied the invertebrate fauna of Dim Cave in Antalya Province, a site under intense anthropogenic pressure from tourism, and Kunt et al. (2010), who published the first checklist of Turkish cave invertebrates.

Three of the species described here were collected from Ayvaini and Great Oylat Caves, two natural karst systems in the Marmara region, where records of cave-dwelling spiders remain scarce, with previous studies documenting only a few species from a limited number of caves in Bilecik, Istanbul, Tekirdağ, Kırklareli, Bursa, and Yalova (Roewer 1959; Brignoli 1968, 1971, 1972, 1978a, 1978b; Topçu et al. 2014; Demircan and Topçu 2015, 2017; Huber 2020). Ayvaini and Great Oylat Caves are particularly affected by high levels of human activity. The recent expansion of cave tourism, accompanied by intense lighting, noise, and visitor pressure, has caused noticeable declines in populations of previously recorded species, making the collection of adult specimens increasingly difficult (Kaya, pers. obs.). As a result, the spider assemblages of both caves may already be changing, even before their diversity has been properly documented.

## Supplementary Material

XML Treatment for Cybaeus anatolicus

XML Treatment for Dictyna caligaformis

XML Treatment for Drassodes robatus

XML Treatment for Drassodes

XML Treatment for Pterotricha kochi

XML Treatment for Cataleptoneta aydintopcui

XML Treatment for Leptonetela

XML Treatment for Leptonetela ayvaensis

XML Treatment for Leptonetela oylatensis

XML Treatment for Troglohyphantes

XML Treatment for Troglohyphantes karolianus

## Footnotes

For a list of five other taxonomic entries, see WSC (2026).

## Additional information

### Artificial Intelligence (AI) use

The authors accept full responsibility for the content of the manuscript, including the disclosure of any use of AI.

No AI tools were used in the preparation of this manuscript.

### Author ORCIDs

A. Zamani https://orcid.org/0000-0002-8084-9666

Y.M. Marusik https://orcid.org/0000-0002-4499-5148

R.S. Kaya https://orcid.org/0000-0002-3769-9105

### Data availability

All of the data that support the findings of this study are available in the main text.
